# Extra Supplement—The Nursing Mirror

**Published:** 1890-12-13

**Authors:** 


*The Hospital*, December 13, 1890. *Extra Supplement.*

### "The Hospital" Nursing Mirror.

Being the Extra Nursing Supplement of "The Hospital" Newspaper.

All Contributions for this Supplement should be addressed to the Editor, The Hospital, 140, Strand, London, W.C., and should have the word "Nursing" plainly written in left-hand top corner of the envelope.

### En Passant.

AT HULL.—An institution of trained nurses for visiting the suffering poor, under the direction of the medical men, in the poorer districts of the town, has just been formed by Mrs. Goodwin, of the Nurses' Home, under most favourable auspices. This lady has already, by special request, organised a similar staff for district visiting at Gainsborough, both institutions being worked on an unsectarian basis.

LEPROSY NURSING.—Miss Marsden expresses to friends at St. Petersburg her concern at what she saw at Constantinople of the pitiful condition of the lepers. Not being allowed into the town, they dwell wherever they can find shelter, and numbers live in the great cemetery at Scutari. It is very sad to think that there is no hospital accommodation for such sufferers. Miss Marsden believes that if the subject were thoroughly ventilated the Turkish Government would take some action. She believes they would even follow the example of the Russian Government, who are talking of building an hospital in the neighbourhood of Riga for the lepers in the Baltic Provinces.

NURSING IN LUNATIC ASYLUMS.—We shall publish next week a letter from an attendant in a private asylum, who is a constant correspondent of ours, and who works where there is excellent training for the attendants. Last Saturday's *British Medical Journal*, in reference to the subject of sick nursing in lunatic asylums, points out that the number of sick in asylums is very small, about five per cent., and that mental nursing needs different training to ordinary sick nursing. The contention is that the hospital nurse should not enter the asylum, but that asylum attendants should be taught sick nursing. But, then, how are the attendants to be taught sick nursing unless there be one trained nurse at the head of the infirmary ward to teach the attendant under her. The article concludes: "Physical strength and courage are essential in the making of a good asylum nurse; given these, with the training proposed, add the refinement of feeling and wider sympathy we all desire to see brought into the service of the insane, and we expect that the asylum nurse of the future will compare favourably with those engaged in any other department of nursing."

SHORT ITEMS.—Mr. Henry Irving has placed a box at the Lyceum at the service of the nurses of the Royal Free Hospital for a certain number of nights.—Ward maids have been instituted at the Middlesex Hospital; they do not live in the building, but come on duty every morning at six a.m.—Two new homes in connection with the Liverpool Training Home will be opened in January.—Princess Louise (Marchioness of Lorne) and Princess Beatrice (Princess Henry of Battenberg) last week attended a meeting of the council of the Queen Victoria's Jubilee Nurses' Institute at St. Katherine's Hospital, Regent's Park. Princess Christian of Schleswig-Holstein, who is a member of the council, was also present.—The Brassey Holiday Home, since it was started in May, has been visited by almost 200 nurses. Many of the visitors speak highly of the pleasant time they spent in the home.—Some nurses in uniform attended the ball in aid of the Royal Free Hospital, and attracted much attention.—The Superintendent of Nurses at the John Hopkins' Hospital, Baltimore, receives fifteen hundred dollars a-year.—Three new branches of the Queen Victoria Institute are to be opened in Scotland this month.

NURSES' CONVERSAZIONE.—A pleasant gathering of nurses was held at Princes' Hall, on December 5th, under the auspices of the British Nurses' Association. The galleries were crowded with nurses in every variety of uniform, those of St. Bartholomew, the Royal Free Hospital, and the Golden Square Throat Hospital, being the most numerous; there were also a good number of Middlesex and Great Ormond Street sisters and nurses present. Guests were not so numerous as last year, and visitors from the country were fewer; there was one Army sister, and one or two matrons from infirmaries; Miss Thorold received the guests as they arrived. It was very pretty to see Princes' Hall about ten o'clock, when Corney Grain was expected; both below and in the gallery it was crowded with bright-faced nurses, who seemed to have thrown from them, for the time being, every remembrance of the sick room; they were all laughing and chatting and eating ices, and generally enjoying themselves. A telegram was read from Princess Christian, wishing all present a happy evening, and then the old favourite—Mr. Corney Grain—stepped on to the platform, to be greeted with applause and laughter before ever he opened his mouth. For about half an-hour he kept his audience highly amused by his musical sketch of a trip to a watering place, and then the meeting began to break up and the nurses to disperse to their various hospitals.

A WOMAN'S VIEW.—It appears that Mrs. Hunter considers herself aggrieved because of the scant courtesy with which she was heard at the meeting of governors of the London Hospital last week; as a woman standing up alone to face some 200 men in utter opposition to her, our sympathy and admiration are with Mrs. Hunter. But is one's sex any excuse for ignorance, which perpetrates untold evil on the sick poor of East London? Mrs. Hunter has suddenly taken up a cause—the cause, as she believes, of overworked nurses. Mrs. Hunter rushes into print, raises accusations, manages to impress on part of the public suspicions about the management of the London Hospital. The committee of the hospital, the governors of the hospital, the medical staff of the hospital, the nursing staff (past and present) of the hospital, all point out to Mrs. Hunter the fallacy under which she is labouring, they also try and point out to her how she is being misled. But Mrs. Hunter, who knows nothing of hospitals or nursing, does not pause in her headlong career; though the charges she brings forward are disproved she reiterates them. Some of the mud begins to stick, and the patience of all fails them before this illogical and unjust attack and cries and groans greeted Mrs. Hunter at her last attempt to produce discord and disaster at the London Hospital. Mrs. Hunter believed she acted for the nurses, but where were the nurses for whom she acted? The supposed over-worked, under-fed nurses of the London Hospital were grouped round their matron, turning indignant eyes, and hurling scathing remarks at the woman self-appointed their champion. No finer sight of woman's loyalty and fealty was ever seen than the way in which the nurses of the London Hospital have upheld Miss Lückes during the late struggle. Letters, testimonials, presentations were showered on their beloved matron; no company of men ever stood by their captain more truly than these nurses by their matron; all honour to them, and all hearty congratulations on the great victory won partly by their steadfastness and truth.

### The London Hospital and its Nurses.

The following is the report of the Sub-Committee appointed on the 14th of October, to report to the House-Committee of the London Hospital, on the allegations which have been recently made against the nursing department. The report is slightly condensed to enable us to let it all appear the same week.

The allegations were five in number, and are given below in their proper order, with the answer of the Special Committee of the Hospital.

1. *That too much power is entrusted to the matron with regard to the dismissal of probationers for inefficiency.*

The scheme of management which the House Committee has adopted, so far as the Nursing Department is concerned, is in the main that recommended by Miss Nightingale in her memorandum handed in to the Lords' Committee, and accepted and issued by them with the evidence. The following are the most material passages:—

"I may perhaps again point out that the Superintendent should herself be responsible to the constituted hospital authorities, and that all her nurses and servants should, in the performance of these duties, be responsible to the superintendent only.

"No good ever comes of the constituted authorities placing themselves in the office which they have sanctioned her occupying.

"No good ever comes of any one interfering between the head of the nursing establishment and her nurses. It is fatal to discipline.

"All complaints, on any subject, should be made directly to the superintendent, and not to any nurse or servant.

"She should be made responsible, too, for her results, and not for her methods.

"Of course, if she does not exercise the authority entrusted to her with judgment and discretion, it is then the legitimate province of the governing body to interfere, and to remove her.

"It is necessary to dwell strongly on this point, because there has been not unfrequently a disposition shown to make the nursing establishment responsible on the side of discipline to the medical officer, or the governor of a hospital.

"Any attempt to introduce such a system would be merely to try anew, and fail anew, in an attempt which has frequently been made. In disciplinary matters a woman only can understand a woman."

The London Hospital Scheme, in so far as it differs from that of Miss Nightingale, differs in restricting, to some extent, the matron's powers.

In these recommendations of Miss Nightingale's we entirely concur. It would be unjust, as we think, to throw upon the matron the whole responsibility for the nursing staff, and at the same time to withhold from her the authority necessary to enable her to secure proper discipline, and the due efficiency of the staff.

Those who think it dangerous to entrust the matron with the power of dismissing probationers for inefficiency, do not, in our judgment, give sufficient weight to the safeguards which exist against the unfair exercise of that power.

The last thing the head of the nursing staff can desire is to part with an efficient probationer. Again, the matron is not, as is the case in our households, brought into constant personal communication, and possible personal disagreement, with her subordinates. She dismisses, if at all, unwillingly, uninfluenced by personal bias, and not until repeated trial under different sisters has shown the probationer to be incompetent. A young woman may be irreproachable in conduct, and may give "no cause for complaint," yet she may be unfitted for hospital nursing, and must give place to someone more competent, otherwise the standard of nursing would be lowered.

We willingly admit that the practice of other hospitals affords no justification for abuses in our own. At the same time, as the London Hospital has been selected for special attack, and the abuses alleged to exist are, by inference at least, alleged to exist exclusively in that hospital, we have caused enquiries to be made of the other leading London hospitals, and of the Edinburgh Infirmary, with the following results:—

In ten out of the eleven hospitals (including the London) the power of dismissing the probationers is vested in the matron, subject in the case of two hospitals (of which the London is one) to an appeal to the Committee; in one, questionable cases are referred to the Committee; in one case the matron dismisses "subject to the approval" of the chairman of the Sub-Committee; and in another "subject to the approval" of the Medical Superintendent.

In five cases (including the London) a report of each dismissal is made to the House Committee.

It thus appears that, with hardly an exception, the other hospitals have found it necessary to vest in their matrons the power of dismissal, which, in the case of the London Hospital, has been announced as grossly unjust.

The extent to which the power has practically been exercised in the London Hospital, appears from the following return:—

The total number entered on the registers as regular probationers from November, 1880, to November, 1890 (a period of 10 years), was 599; 37 were compelled to give up by health, 25 left on grounds of health (reasons probably mixed), six left to be married, 31 deliberately broke their engagement, 20 were by the committee discharged or allowed to resign for misconduct, 13 broke engagements with mutual consent, three died, and eight engagements were terminated by the matron on the grounds of inefficiency and unsuitability for training. These probationers had prolonged trials, varying from 5 to 12 months.

In only eight cases out of 599, therefore, has the matron's power of dismissal been exercised. In every case the dismissal was reported to the committee before the probationer left the hospital.

Our conclusion is that in so far as it implies that the London Hospital system is especially open to criticism, as being unjust to the probationers, the allegation is itself unjust, and that no serious grievance exists.

2. *That with regard to the Private Nursing Institution, insufficiently trained nurses are withdrawn from the wards and sent out as thoroughly trained nurses.*

In considering this question, it is obviously necessary to determine what constitutes a trained nurse, and it is important to bear in mind the difference between a "trained" and a "certificated" nurse. Much confusion and misapprehension has been occasioned by treating the two as identical.

The following information, obtained from the hospitals to which we have already referred, illustrates what we mean.

In nine out of the 11 hospitals, a nurse is considered "trained" in one year.

In one case (that of St. Thomas's) no certificate is given. In two cases (the London being one) a certificate is given at the end of two years. In the other cases at the end of three.

The Army and Navy nursing services require not three years' training, but three years' service. Thus, in hospitals where there is only one year's training they require two years' service, and in hospitals where two years' training is the rule, one year's service.

At the London Hospital a nurse is not permanently put in charge of a ward until she has had two years' experience, and is certificated. In six of the other hospitals she is eligible for such an appointment at the end of one year although uncertificated and in every case she is eligible before the expiration of two years or three years, as the case may be, great care being of course taken to select only such probationers as are fitted for the appointment.

It will be seen therefore that a trained nurse and a certificated nurse are by no means identical. The truth is that one nurse may be more thoroughly trained at the end of twelve months than another in two or three years.

All the members of the private nursing staff, are certificated nurses.

During the whole period of five years 162 nurses have been sent out without being on the private nursing staff at the time of being first sent to a case. Of these, 35 subsequently joined the private nursing staff.

Out of the total number of 162, 24 had had training and experience in this hospital for periods ranging from two to nine years. Out of this total number of 162, 33 only have been sent with less than one year's experience in this hospital, and 19 of these had had previous experience elsewhere. Out of the remaining 14, nearly all were sent by special request.

The total number of cases supplied with nurses from January, 1886, to October 24th, 1890, is 1,409, and out of this large number there have been unsatisfactory reports in only seven cases.

The rule is that a nurse is never withdrawn from a ward and sent to nurse a private case unless she can well be spared. She is frequently, as already stated, sent by special request, and often because it is found that a nurse's health benefits by the temporary change where the case is at the seaside or some healthy country-place.

That out of this large number of cases there should have been so few complaints of any kind s eems to us to be strong evidence in favour of the London Hospital private nursing system.

The facts above stated seem to us to afford a complete answer to the second allegation.

3. *That the nurses' food is insufficient and unsuitable.*

Formerly, complaints by the matron, as representing the nursing staff, were frequent. These complaints were not so much of insufficiency of the food as of the way in which it was cooked and served; and many attempts were made to cure the evils complained of. After the establishment of the Nurses' Home, the matron took charge of this department. She administers it with the help of one of the sisters, who is responsible to her, and although there have been occasional complaints (as for instance recently, where the House Committee found it necessary to cancel the contract for the supply of meat which had just been made with a new contractor), we believe the system works well. It has been the practice of some of us, when acting as visitors, to visit the dinner table, taste the food, and inquire of the nurses if they had any complaints to make. From our own observation, as well as from inquiries we have made, we are satisfied that the food is not only abundant but well served. It will hardly be expected where so large a number has to be fed that the taste of each individual can be considered, but those who are responsible for the food are thoroughly alive to the importance of its being not only nourishing and sufficient, but also appetising. The dietary of each day and night is varied, the particulars are recorded on forms supplied for the purpose, and signed by responsible sisters, who at the same time record any complaints they receive. [Note.—The bill of fare selected for special criticism was served on a Friday. On that day, out of consideration for the Roman Catholic members of the nursing staff, fish forms an important item.]

These returns are inspected each day by the matron, and the system affords, we think, effectual security that any weak point will be discovered, and we see no reason to suggest any alteration.

Special objection has been made (as it appears to us under a misapprehension) to the meals served to the night nurses. With them day is turned into night. Like ourselves, they begin with breakfast before going to their work. This is served at 8.50 p.m., and the meals which are served would be considered sufficient and acceptable at our own tables. Between 12 and 1 they have their ward meal, which they have taken with them into the wards. At 5 a.m. they have tea and toast, which answers to our five o'clock tea, and at 10 o'clock in the morning, after their work is over, they have their dinner. In addition, milk is always available for those who require it.

4. *That the staff is insufficient, that too great responsibility is thrown upon nurses not thoroughly trained, and that there are too many paying probationers.*

Here again some confusion has arisen from treating "certificated nurses" and "trained nurses" as identical, and as to this we would refer to our remarks in answer to the second allegation. It will be seen, that in the case of every other hospital, nurses are eligible for appointment to the charge of a ward who are not "certificated," and who, therefore, would be classed as "untrained," if the same rule were applied to them as our critics seek to apply to the London Hospital.

As the matron points out, a sister of a ward must not only be a good nurse, but she needs all the qualifications of the general head of a household to be a really good sister. A mere certificate is no test of such qualifications, which belong naturally to some of comparatively short experience, and can never be acquired by others, however long their period of training may be. Great care and judgment in selecting nurses for sisters and for staff duty is of all things important.

It must not be forgotten that as the permanent staff has to be recruited from the probationers, it is important to have a large number from which to select, and important, we may add, in the interests of the probationers as well as the hospital, to throw upon them (of course within proper limits) as much work as they can fairly do. It is in this way they are thoroughly trained for staff appointments, and it is because the London hospital probationers are known to be thoroughly trained in varied and practical work, that they stand so high in the estimation of the nursing world.

Objection is made to the number of paying probationers who leave at the end of three months. These last have been 279 only in a period of nearly nine years. The number of paying probationers cannot be ascertained by dividing, as our critics have done, the profits for any year by 52, because the same individual in many cases pays many times over.

Many of these paying probationers had had considerable nursing experience before entering the London Hospital. All enter from a love of the work, and the great majority are willing workers, anxious to learn. The rule of the hospital is that no distinction as regards work is made between paying and regular probationers, except that the former are not required to do night duty. There are many ways in which valuable help can be rendered, even by inexperienced nurses, and it is a fact that many of the most efficient members of the staff began their career as paying probationers.

The number of paying probationers, whether or not they have renewed their terms, is limited to 30, and in practice the average number is much less. They render good service to the hospital which, moreover, derives from them, and especially from those, who renew their term, a not inconsiderable revenue.

We find, as indeed might be inferred from these figures, that the paying probationer system works well, and that the suggested grievance is theoretical only.

As regards this fourth allegation, as well as others, our critics have been led into the error of drawing general inferences from exceptional cases, in many of which, moreover, the facts are disputed, and others of which occurred under circumstances altogether different from those which now exist.

Efficient help is always within reach, if needed, either in the day or night. No inexperienced nurse is left in responsible charge of serious cases. The number of nurses required depends upon the number of severe cases. No hard and fast rule can be laid down, and one great merit of our system is its flexibility. We are assured that when the cases are heavy, the proportion of one sister, two staff nurses, and two probationers by day, and one staff nurse and two probationers by night, to every 30 beds, which is the matron's ideal number, is frequently provided.

The system instituted by the matron, under which each head is responsible for her subordinates, and each sister daily makes a full report to the matron of all that has occurred in her ward, and consults with her when necessary on any question of management, secures effectual supervision, and creates in the whole staff a due sense of responsibility. The result is that, according to the unanimous testimony of the medical and surgical staff (than whom no one is better qualified to judge), the nursing is thoroughly efficient.

In our judgment the grievances alleged under the fourth head have no practical existence.

5. *That too much menial work is cast upon the nurses, that the hours are too long, and generally that the nurses are overworked.*

It is not seriously alleged that in consequence of over-work the patients suffer; indeed, more than one of the witnesses expressly states the contrary. The charge is that the nurses are sacrificed to the patients.

There can be no doubt that in times of pressure the work is arduous and such as cannot be adequately performed except by persons specially fitted for it. The sisters especially have very hard work and great responsibility thrown upon them, owing very much to the plan of the hospital building, and the large size of some of the wards. Fortunately, times of pressure are often followed by quiet times, and after a heavy week the nurses frequently have extra times of four hours given them daily, and always before six o'clock p.m., so as to allow of their getting air and exercise.

Whatever the work may be, the duties of the nursing staff are discharged with a self-devotion which deserves our highest praise and admiration.

The greatest possible care is taken with regard to the nurses' health. On all hands we receive testimony to the exceptional thoughtfulness and kindness of the matron in every matter relating to health. The sisters report to her every case in which the health of a nurse appears to them doubtful, and the rule is that in any such case she shall see either Dr. Sutton, Dr. Fenwick, or Mr. Treves, members of the senior staff, who have kindly undertaken the special care of the nurses. The cases which have been referred to to show that the mortality in the London Hospital is exceptional and due to overwork are misleading. We have been unable to trace any such case. The majority of the deaths which unhappily have occurred (and the number has been 16 in 10 years, out of a total number of 1,165 workers) were due to organic diseases; others were the direct result of contact with patients they were nursing, and of these three died of diseases they had contracted from the patients they were nursing outside the hospital, and three others, although included in our list, died away from the hospital.

Complaint is made of the hours of work, but the division into two shifts day and night is, according to universal opinion, inevitable. It is adopted in every one of the hospitals to which we have referred, where the hours of work are in the majority of cases practically the same as in the London Hospital, differing in almost every case where they do differ in favour of our own hospital. It is the practice at the workhouse infirmaries, at the county lunatic asylums and Metropolitan Asylums Board institutions, where the anxieties and work are certainly not less than at the London Hospital.

Especially our system with regard to the hours off seems to us to be the best. All our nurses and probationers get two hours, and very often four hours, off duty daily—and, what is very important, before six p.m. Many of the other hospital give the hours off on alternate days only, and many give them too late to allow of young women going out for fresh air and exercise.

Much difference of opinion seems to exist as to the proportion the number of patients should bear to the number of nurses. The London Hospital compares favourably in this respect with all the other hospitals, and especially as regards the proportion of night nurses to patients. We repeat that in referring to the other hospitals, our object is not to cast blame on them or to excuse any shortcomings of our own; our object is to show the injustice of selecting the London Hospital for special attack, and also because the facts we have stated show either that the governing bodies are, on the whole, satisfied with the existing state of things, or that there are practical difficulties in the way of any material alteration of their system, which they have not seen their way to overcome, although some of them are much more richly endowed than the London Hospital. In our own case, any material increase in the staff means necessity for providing increased accommodation on a large scale, and means consequently increased expense, which the hospital is especially ill able to afford at the present time, when the large sum necessary to carry out the sanitary improvements has to be provided. The alternative of closing some of the wards would be regarded by the district, and we believe by the nurses themselves, as a calamity.

We would suggest, as one means of relieving the nursing staff to some extent, that, following the matron's recommendation, the number of ward maids should be increased. This would not necessitate the providing of additional accommodation within the walls of the hospital, and it would relieve the probationers from the discharge of the menial duties now performed by them for the sisters, and which, although they are much diminished of late years, and are, as we are informed, discharged by the majority willingly, and even eagerly, are by others considered as a grievance.

It is so important that the lamps should be in perfect order that the cleaning of them, which has been complained of as a grievance, must be done by the nurses or by some skilled person. It is worth consideration whether one man should not be made responsible for cleaning and trimming all the lamps—as we are informed is the practice at St. Bartholomew's.

### General Summary.

Throughout our inquiry we have had in mind the importance of calling attention to every weak point, suggesting alteration in our nursing system, or its administration. As we have already said, the nursing department is organised mainly upon the principles recommended by Miss Nightingale, and we see no reason for recommending a change.

Of course, mistakes may, and will, occur under any system. During the last 10 years no less than 76,985 patients have been admitted as "in-patients" to the wards of the hospital, and there have been 1,165 of the nursing staff entered on our books. That so few mistakes should have been made seems to us strong evidence in favour of our nursing system. Its successful administration depends, of course, mainly upon the efficiency and trustworthiness of the staff—and especially of the matron. Here it is that our critics are in entire disagreement, not only with the House Committee, but with the whole medical staff, all the sisters, and a large majority not only of the present nursing staff, but, so far as we have been able to ascertain, of such of its former members as had the advantage of working under Miss Lückes, and, we may add, of the nursing world generally. There is no disguising the fact that the present attack is directed mainly against our matron. She is charged with neglecting her duties, neglecting, underfeeding, and overworking the nursing staff, and arbitrarily and unjustly dismissing the probationers. These charges are, for the most part, based on information derived from persons who cannot pretend to any but elementary hospital experience. To anyone who knows the matron, and what she has done for the hospital and the nursing staff, such charges must appear altogether extravagant and incredible.

During the 10 years of her service the nursing has been entirely reorganised, the nursing staff has been largely increased, the nursing home has been established, a nurses' pension scheme has been adopted, a sound system of theoretical training has been established, the hours "off-duty" have been increased, the holidays have been doubled, and generally the nursing and the condition of the nursing staff have been improved to an extent which only those who knew the hospital before Miss Lückes became its matron can thoroughly appreciate. In short, the history of the 10 years of her management of the nursing department is the history of constant improvement, and of constant and practical solicitude for the welfare of the nursing staff.

What has been done in the last 10 years may, we think, fairly be taken as an earnest of what will be done in the future, as opportunity arises. We are satisfied that the system is thoroughly sound, that on the whole it works admirably, and that such alterations as may be desirable are in what may be called matters of detail only.

The governors may rest assured that there is an anxious desire on the part of those responsible for the welfare of the nursing staff to promote their health and well being in every possible way, and that any complaint made with the honest desire to improve the working of the hospital will receive—as every complaint always has received—careful consideration, with the view of remedying any abuse which may be proved to exist; but we cannot doubt that the House Committee, knowing what they do, would regard it as in the highest degree ungrateful and unjust to ignore the great services which the matron has rendered to the hospital, or consent to any step which would imply a want of confidence in her, or tend to weaken her authority.

### CONSOLATION.

"Now let us thank the Eternal Power; convinc'd
That Heaven but dries our virtue by affliction,—
And oft the cloud which wraps the present hour
Serves but to brighten all our future days."

### Everybody's Opinion.

[*Correspondence on all subjects is invited, but we cannot in any way be responsible for the opinions expressed by our correspondents. No communications can be entertained if the name and address of the correspondent is not given, or unless one side of the paper only be written on.*]

### FEVER HOSPITALS.

"THISTLE" writes:—Some of our provincial fever hospitals are sadly in need of a clean sweep, although I did not imagine any hospital to be so far behind the times as one I had occasion to visit the other day. One of our district patients—an old lady—had been removed to the fever hospital, and at the earnest request of her son, who was also lying ill, our lady superintendent went to visit her, and asked me to accompany her. We had some distance to walk before we came to the hospital—rather a nice building outside, which is all we saw. As we entered the gate, two women were standing on the hall steps, one a visitor, the other, I thought, was a very untidy wardmaid. Imagine my surprise when, dismissing the visitor, she turned to "Sister" and shook hands, saying, "I suppose you have come about that old woman you sent up yesterday—no, the day before. Well, she's very bad, can't see you; she can't live, poor thing. It's a pity." "But," interposed Sister, "her son wished so much for me to see her, and the doctor said there could be no objection." "Well, you can't—you can do her no good—only upset her. I asked her, and she said she did not want to see you. We've sent for her daughter to come and help sit up nights with her. I'm sure you're very busy—so am I. It's a nice day, very," and with a poke outwards with her hand went inside and closed the door, leaving us standing on the steps. Now what manner of matron is she to have charge of people "sick unto death," or to train women to be good nurses? Her own appearance, in the first place, would need looking to before she could speak to her nurses on tidiness. When I saw her she had on a dirty print wrapper, with several buttons wanting; no cap, collar, nor cuffs, looking altogether more like a charwoman than matron. I have since heard that some of the nurses go on duty wearing silk, grenadine, &c., dresses; so long as they have on an apron they are all right. Is it any wonder that many of our poor patients prefer the small chance of getting better at home to going to the Fever Hospital, after hearing the sad story of the poor old lady? Her son, who was in lodgings, was taken ill with enteric fever. His mother came to see him, and after a few days she, too, was very ill. There being seven people for one room, it was thought advisable to remove her to the hospital. Two days after she died, without having seen any of her relatives, and, indeed, none of them were informed of her death until the next evening, although in the afternoon a child had been up to inquire for her. When her friends arrived at the hospital to make arrangements for the burial, they found she was just about to be carried out, and so were just in time to prevent it, until some of her relatives were communicated with.

### HELP WANTED.

THE MATRON of the Bristol Hospital for Sick Children and Women, St. Michael's Hill, writes: I venture to ask you kindly to allow me space in your columns to solicit the help of our benevolent friends, that the approaching season of Christmas may be a bright and merry one to my little patients. Children occupied a large portion of the large heart of the Master; sick ones were specially cared for. Did He not say, "When thou makest a feast, call the poor, the maimed, the lame, and the blind; and thou shalt be blessed; for thou shalt be recompensed at the resurrection of the just"? I feel, therefore, I can with confidence plead for the little sick ones who will be spending their Christmas within the wards of this hospital, which we desire, as usual, to make particularly bright. I shall be glad to receive anything that kind friends may feel disposed to give. I hope to get six large Christmas trees for the general wards, and any kind friends wishing to present trees will do a great favour by informing me not later than the 11th inst. Plants, evergreens, and other decorative material will be welcomed. Those who have little ones of their own know that "lollies," oranges, and suchlike are in immense favour with our juvenile population; nor do they in any wise despise such things as dolls, toys, books, &c. But while speaking of the things that delight the eye, the mind, and the palate, I would also mention those that are necessary for maintaining the heat and comfort of the body, and shall be very glad to accept woollen or any other articles of clothing, as well as boots and shoes. Before closing, I would speak a word for the nurses. The vocation of a nurse necessarily involves self-sacrifice; and in many cases the love and tenderness belonging to, but lacking in, the parents are for the time supplied by the nurse. Some kind friends will, I am sure, give special thought at this season for the nurses who so cheerfully minister to the necessities of their little charges.

### A PRACTICAL HINT.

"MARGUERITE" writes: Might I ask you to point out, through the medium of your paper, a fact that does not seem to be generally known, as Dr. Miles in his "Lectures on Surgical Ward Work and Nursing" does not mention it, viz., the great superiority of mercurialised glycerine, 1 in 500, over carbolic oil or eucalyptol for lubricating and rendering antiseptic catheters, bougies, speculars, &c. The hyd. perchlor. remains always antiseptic, being non-volatile, and the glycerine does not become stale in the way oil does. I have used it for some years, and find it very slightly destructive to instruments.

### THE TRAINED NURSE IN THE NURSERY.

"A LOVER OF DUTY" writes: I have been very much struck by a letter from "Sister Grace" in the "Nursing Mirror" for November 29th. It takes a thorough grip of the subject, and is marked by sound common sense throughout. Her motto, like Nelson's, is obviously "Every man is expected to do his duty." This she infers when she says a nurse should be "strictly truthful, honest, clean, discreet, and respectful." What mother or any other human being could desire more? But nothing less than a strong sense of duty, built on a good religious foundation, will ensure all these excellent qualities. She touches on another crucial point when she says nurses are frequently above their work. "Jack's as good as his master," and rather better, is, unfortunately, a sentiment which permeates society nowadays. It used not to be so. I myself have known many who have been faithful and true servants to both their earthly and heavenly masters. One lived forty years in our own family, bringing up two generations of children, and she was in all points irreproachable. She became a factotum in the household, and when pensioned off remained a cherished friend till her death, in her ninety-second year. She could read, write (her spelling was phonetic), superintend and do housework, cook in a most toothsome way, and her needlework was excellent. What modern nurse, with all her nineteenth century advantages, is her equal? But she had been taught to "love, honour, and obey," and she acted up to her lights, and made her employer's interests her own. All nurses are the better for a genial, sympathetic nature, and would it be quite out of place to hint here that sick, especially hospital, nurses are apt in press of work to forget that a patient is a human being, and to treat him as a case. They should try and realise the misery and discomfort of being moved in racking pain from home, with its familiar surroundings, and planted in a large, strange ward, with the publicity attending such a proceeding, when all that the poor sufferer wants is to hide like a stricken deer from his kind. It is pardonable in a patient to be fretful or irritable or low-spirited at such times, but if the nurse could "put herself in his place" at first, and be pitiful and courteous, as St. Peter directs, it would much add to the comfort of both in the end.

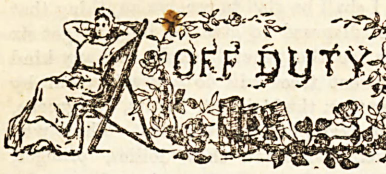

### Domestic Medicine in the 18th Century.

*(Concluded from page lii.)*

The efficacy of many other receipts are vouched for, *i.e.*, "This cured an acquaintance of mine, who told it me himself." One for pleurisy is "approved by myself." "Four spoonfuls of brandy" (size not notified) cured Mr. Blundel, of Hampstead, of colic—O shade of Sir Wilfrid Lawson!—"after he had had the advice of several able physicians, and had been at the Bath without success." A quinsy in the throat was to be thus treated: "Shave the top of the head, and bind on it, with a cloth, a toast of household bread well baked on both sides, and soaked in right French brandy." "If this be done at night, going to bed, it will cure before morning, as I myself have had experience of. S.C." What can be said for ground-ivy heated between two tiles and "lain as warm as can be borne" on the top of the head? Would it draw up the uvula nowadays?

There are several receipts for the bite of a mad dog, from "Primrose roots steeped in white wine and strained, let the patient take a good draught," to a more elaborate one which requires a whole page of letter-press for its narration. An "infallible cure" has the following N.B. affixed: "This receipt was copied out of Cathorp Church, in Lincolnshire, the whole town almost being bitten, and not one person who took this medicine but was cured," *mirabile dictu!* Could it have been that the dog was the identical dog immortalised by the poet Goldsmith, who sings, "And strange to tell the man got well, The dog it was who died"?

A singular efficacy must have resided in egg-shells, oyster-shells, snail-shells, and in powdered shells of all kinds, and, oh! horror, in snails themselves, since those slimy and repulsive creatures figure largely in most of the prescriptions. Possibly they were used so freely that the supply could not meet the demand, and hence the decline and fall of many remedies. Gardeners might now be depended on for having a few at their disposal should anyone be desirous of trying their curative powers. In simplicity of treatment and success in the cure of rheumatism and consumption the receipts of the "Compleat Housewife" throw Sequah into the shade, and Dr. Koch is nowhere. Why, then, should such valuable knowledge have been forgotten? Echo answers, Why?

### Notices.

Several important matters have been unavoidably held over this week.

Would our correspondents kindly send us brief accounts of their Christmas festivities (written on one side of the paper only) at the earliest date? We should like to get as many of these as possible into our New Year's Number; to achieve this they should reach this office by December 27th.

About thirty answers have been received to the last examination question; the award will be made next week.

All bed-jackets and Nightingales for the new competition must reach the Hospitals Association, Norfolk House, Norfolk Street, Strand, London, by Wednesday, December 17th.

### Appointments.

[It is requested that successful candidates will send a copy of their applications and testimonials, with date of election, to The Editor, The Lodge, Porchester Square, W.]

Devizes Borough Hospital.—Miss Esther Jones, night superintendent of the Monsall Fever Hospital, Manchester, has been appointed to the charge of this hospital.

Indian Service.—Nurse A. J. Weighell, who has just finished her training at the Bristol Royal Infirmary, has been appointed by Lady Roberts to the post of sister at the military hospital at Quetta, India.

Luton Cottage Hospital.—Nurse Babcock, of St. Thomas's Hospital, has been appointed matron of this institution.

### Notes and Queries.

### Queries.

(20) *Percentage.*—Is it possible to learn how much percentage on earnings is given to the nurses in some institutions? We want to do something of the kind for our staff, and shall be glad of any information.—*Felix.*

(21) *Work for Incurables.*—Wanted, the names and addresses of societies which supply work for incurables.—*F. A. B.*

### Answers.

*First Thousand Nurses.*—Excellent oak frames for certificates can be procured at a cost of 3s. 6d. each, packed ready for delivery, from the City Frame Company, 29, Basinghall Street, E.C.

*One Interested.*—The delay is necessary, you will receive particulars later on.

*N. S.*—Dr. Dutton's pamphlet we have not got, and we do not know the name of the publisher, but the whole gist of the pamphlet was given in the article in The Hospital. The main point was to take a blue pill and effervescing draught a fortnight before going to sea; to repeat the treatment a week later, and to live on a restricted diet, avoiding stimulants.

(12) *Temporary Home Wanted.*—Apply to Dr. Phillips, St. Anne's Heath, Virginia Water, Surrey.

(13) *Starting a Home.*—Your only chance is to secure the support of the doctors; call on all the medical men in the town and consult them as to your proposed scheme.

(17) *Notice.*—It depends entirely under what agreement you took the case; if you are paid monthly, your employers will expect a month's notice.

*Nurse H.*—There is a Nurses' Club at 12, Buckingham Street, Strand, W.C. Open daily from two p.m. Lectures once a-month, a good medical library, and excellent advantages of all sorts. Go and see the secretary.

*M. Ingram.*—A certificate is given after one year at the Chichester Infirmary (fee 10 guineas), Addenbrookes Hospital, Cambridge, Boston Cottage Hospital (fee £26), and Hull Infirmary.

(14) *Asylums in India.*—All asylums in India are under Government control, and enquiries concerning those near Madras should be made to the Secretary of the Madras Presidency.

*Hospital Cots.*—We think "B." will find that Messrs. Brierley and Son's patent hospital cot (No. 3,192), the sides of which and also the end fall down, the one most suited to her purpose.

### Amusements and Relaxation.

**N.B.—THIRD QUARTERLY WORD COMPETITION**

**Commenced Oct. 4, 1890; ends Dec. 27, 1890.**

Three prizes of 15s., 10s., 5s., will be given for the largest number of words derived from the words set for dissection.

N.B.—Word dissections must be sent in WEEKLY not later than the first post on Thursday to the *Prize Editor*, 140, Strand, W.C., arranged alphabetically, with correct total affixed.

The word for dissection for this, the ELEVENTH week of the quarter, being "NATIONS."

| Names. | Dec. 4th. | Totals. | Names. | Dec. 4th. | Totals. |
|---|---|---|---|---|---|
| Jenny Wren | 211 | 592 | Checkmate | — | 76 |
| Tinie | — | 55 | Silver King | — | 163 |
| Agamemnon | 226 | 606 | S. Anthony | — | 76 |
| Patience | 223 | 603 | Quackah | — | 75 |
| Ecila | 225 | 606 | Reynard | 201 | 547 |
| Lightowlers | 219 | 578 | Sally | — | 27 |
| Rouge | — | 89 | Success | — | 61 |
| Wyamaris | 227 | 593 | Caledonia | — | 52 |
| Qu'appelle | 149 | 504 | Nurse Emma | — | 305 |
| Nosam | 180 | 533 | Hazel | — | 20 |
| Nurse Hilda | — | 44 | Pallas | — | 48 |
| Lady Betty | 192 | 552 | Puss | — | 15 |
| Grenelle | — | 43 | Shakespeare | 203 | 490 |
| Daisy | — | 324 | Melita | 195 | 536 |
| H. A. S. | — | 157 | Nora | — | 10 |
| A. B. C. | — | 66 | Elsie | — | 35 |
| Liz | 150 | 417 | Espèrance | — | 27 |

### Notice to Correspondents.

N.B.—Each paper must be signed by the author with his or her real name and address. A *nom de plume* may be added if the writer does not desire to be referred to by us by his real name. In the case of all prize-winners, however, the real name and address will be published.

Competitors can enter for *all* quarterly competitions, but no competitor may take more than *one first prize* during the year.

